# Evaluating the Detection of Hydrocarbon-Degrading Bacteria in 16S rRNA Gene Sequencing Surveys

**DOI:** 10.3389/fmicb.2017.00896

**Published:** 2017-05-17

**Authors:** David Berry, Tony Gutierrez

**Affiliations:** ^1^Division of Microbial Ecology, Department of Microbiology and Ecosystem Science, Research Network Chemistry Meets Microbiology, University of ViennaVienna, Austria; ^2^School of Engineering and Physical Sciences, Heriot-Watt UniversityEdinburgh, United Kingdom

**Keywords:** hydrocarbon-degrading bacteria, Deepwater Horizon, crude oil, 16S rRNA gene, sequencing surveys

## Abstract

Hydrocarbonoclastic bacteria (HCB) play a key role in the biodegradation of oil hydrocarbons in marine and other environments. A small number of taxa have been identified as obligate HCB, notably the Gammaproteobacterial genera *Alcanivorax, Cycloclasticus, Marinobacter, Neptumonas, Oleiphilus, Oleispira*, and *Thalassolituus*, as well as the Alphaproteobacterial genus *Thalassospira*. Detection of HCB in amplicon-based sequencing surveys relies on high coverage by PCR primers and accurate taxonomic classification. In this study, we performed a phylogenetic analysis to identify 16S rRNA gene sequence regions that represent the breadth of sequence diversity within these taxa. Using validated sequences, we evaluated 449 universal 16S rRNA gene-targeted bacterial PCR primer pairs for their coverage of these taxa. The results of this analysis provide a practical framework for selection of suitable primer sets for optimal detection of HCB in sequencing surveys.

## Introduction

**FIGURE 1:**
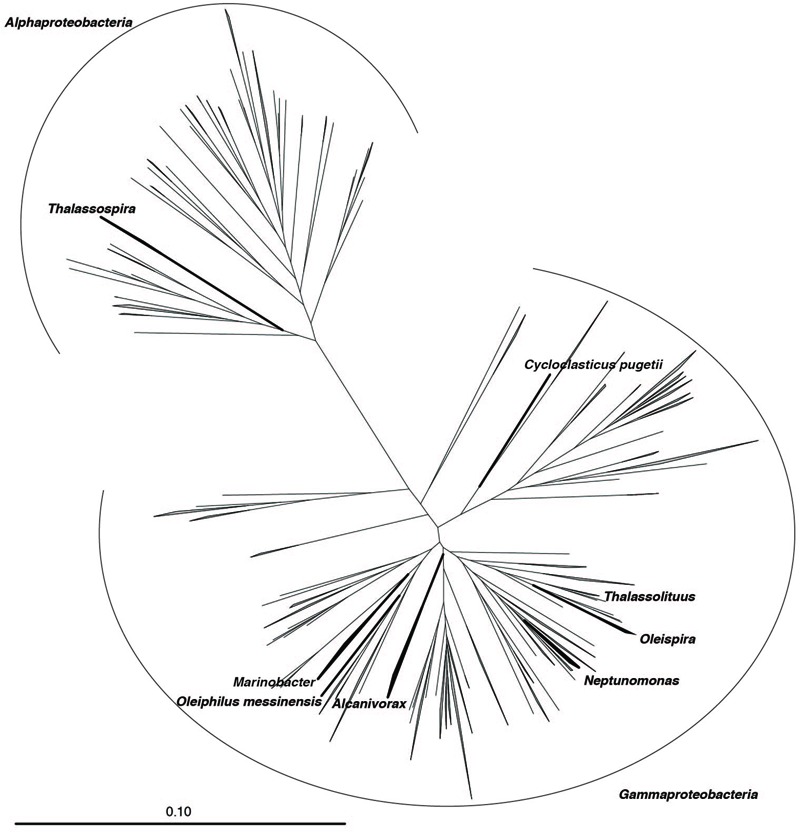
**Phylogeny of taxa with cultivated hydrocarbonoclastic bacteria (HCB) and sister groups.** Neighbor-joining phylogenetic reconstruction of 16S rRNA gene sequences extracted from the SILVA LTP 123 database classified to *Oceanospirillales, Alteromonodaceae, Rhodospirillaceae*, and *Pisciricketsiaceae*. Target taxa are labeled. Bar indicates 10 substitutions per 100 nucleotide positions.

Marine microorganisms play important roles in major biogeochemical processes and in various interactions with higher organisms that, respectively, impact our climate and the sustainability and balance of life on this planet. A subset of the microbiome in the global ocean that plays a fundamental role in the biodegradation and ultimate removal of petrochemical pollutants are the hydrocarbonoclastic bacteria (HCB). The fate of oil in the environment is largely dictated by the presence and activities of these bacteria. Over 175 genera, distributed across several major bacterial classes (*Alpha-, Beta-*, and *Gammaproteobacteria*; *Actinomycetes*; *Flavobacteria-Cytophaga-Bacteroides*), comprise representatives of HCB species (Prince et al., 2010). Most comprise diverse groups of aerobic ‘generalist’ hydrocarbon degraders that can utilize hydrocarbons and non-hydrocarbon substrates as a source of carbon and energy. Generalists may be overlooked when evidence to substantiate their involvement in the degradation of hydrocarbons is lacking. A subset of HCB comprise members that “specialize” in the degradation of linear or branched saturated hydrocarbons (*Alcanivorax, Oleiphilus, Oleispira*, and *Thalassolitus*), or of aromatic hydrocarbons (*Cycloclasticus* and *Neptunomonas*) – i.e., they use these hydrocarbons almost exclusively as a sole source of carbon and energy (**Figure 1**). These fastidious organisms are strongly selected for in oil-impacted environments, where they can increase in numbers from near undetectable levels to constituting up to 70–90% of the total bacterial population, such as during an oil spill (Head et al., 2006; Yakimov et al., 2007). Several other obligate HCB (*Algiphilus aromaticivorans, Polycyclovorans algicola, Porticoccus, hydrocarbonoclasticus*) were discovered in recent years from the phycosphere of marine eukaryotic phytoplankton (Gutierrez et al., 2012a,b, 2013a). The analysis of laboratory and field samples of phytoplankton with CARD-FISH (catalyzed reporter deposition fluorescence *in situ* hybridization) probes, that were designed to target these obligate HCB, has revealed that the numbers of these bacteria on phytoplankton cells is extremely low – from no HCB cells, to one or two detected per phytoplankton cell (unpublished data). This may explain why these organisms are not often detected in sequencing surveys, and their role in the fate of oil hydrocarbons in the ocean remains poorly understood.

The last years have seen a substantial increase in our understanding of marine microbial diversity and function, which has largely been driven by technical advances in sequencing technology that now allow for massively parallelized, high-throughput sequencing and at a manageable cost for most laboratories. The ability to sequence many samples in parallel and to a great depth has offered an unprecedented view on the composition of marine microbial communities. Sequencing-based microbial surveys can generate large datasets that provide insight into microbial biogeography, as well as help reveal microbial community structure and dynamics in response to major perturbations, such as the Deepwater Horizon oil spill. Most of the published literature that investigated the water column microbial community response to the Deepwater Horizon spill utilized conventional (Sanger) and next generation sequencing (pyrosequencing) approaches of 16S rRNA genes (Joye et al., 2014). This revealed the protagonistic role that HCB played in responding to this massive oil spill in sea surface oil slicks (Gutierrez et al., 2013b), in a subsurface oil plume (Hazen et al., 2010; Yang et al., 2016) and in contaminated sediment (Kimes et al., 2013) in the Gulf of Mexico. Assigning metabolic function, specifically the ability to degrade oil hydrocarbons, to the enriched taxa identified in these sequencing surveys was largely based on their phylogenetic affiliation to published hydrocarbon-degrading representative strains. This is common practice in studies investigating the microbial response to oil contamination, mainly because it is convenient and low sequencing allow high-throughput analysis. Other techniques, such as stable-isotope probing, which is an approach ideal for linking taxonomic identity with a metabolic function of interest (Gutierrez et al., 2013b), can be onerous and costly. Cultivation techniques that target the isolation of oil degraders are also highly useful as this allows one to directly interrogate the metabolic potential of isolate strains in the laboratory; however, this can be precluded by the fact that a relatively small fraction of microorganisms are amenable to cultivation in the laboratory.

While amplicon sequencing technologies have advanced significantly in recent years, they rely on suitable PCR primer coverage of target microbial groups (e.g., the HCB) to provide a reliable view of microbial diversity and community structure. To the best of our knowledge, reports describing isolates belonging to any of the genera of obligate HCB have all been shown to degrade hydrocarbons. Hence, we can be highly confident that any organism identified by 16S rRNA gene sequencing to be affiliated to any of the eight genera of obligate HCB will be capable of degrading hydrocarbons. Despite inherent limitations in PCR-based methods such as biases in cell lysis efficiency, differences in rRNA copy number, and variation in amplification efficiency of different templates, PCR-based studies are an important method to catalog microbial diversity. For this subset of bacteria, PCR-based amplification of the 16S rRNA gene remains a powerful tool to link taxonomic identity with the ability to degrade hydrocarbons. In this study, we identified 16S rRNA gene sequences that represent the phylogenetic breadth of key HCB taxa using a detailed phylogenetic analysis. We then used these validated representative taxa to determine the coverage of these groups by a comprehensive list of commonly used universal 16S rRNA gene-targeted PCR primer sets. With this approach, we were able to identify PCR primer sets well-suited for use in sequencing surveys focused on detection of HCB but that also applicable for detecting overall bacterial diversity.

## Materials and Methods

### Processing Sequences for Phylogenetic Analysis

Phylogenetic analysis was aimed at the following taxa, which contain members that are obligate HCB: *Oceanospirillales, Alteromonadaceae, Rhodospirillaceae*, and *Piscirickettsiaceae* (**Figure 1**). These taxa, as well as outgroup genera for them (*Vibrio, Shewanella, Acidomonas*, and *Beggiatoa*, respectively) were extracted from the SILVA SSU Ref 123 database of 16S rRNA gene sequences (Quast et al., 2013). In order to select a smaller number of representative sequences for phylogenetic analysis, extracted sequences were clustered at 97% sequence identity into operational taxonomic units (OTUs) using usearch (Edgar, 2010) and a representative sequence was chosen as the most abundant unique sequence within each OTU. Putative chimeras were identified using usearch and subsequently removed. To ensure that well-characterized strains were included in the phylogenetic analysis, the 16S rRNA gene sequence of type strains from each of the target taxa were extracted from the SILVA LTP 123 database (Yarza et al., 2008) and added to the set of representative sequences. Sequences were aligned with mothur (Schloss et al., 2009). In order to minimize possible artifacts arising from misalignments by the automatic aligner, the resulting alignments were filtered by removing positions in the alignment that were gaps for at least 90% of sequences. Identical sequences (i.e., duplicates) present after filtering of the alignment were de-replicated before phylogenetic analysis.

### Phylogenetic Analysis

Phylogenetic trees from representative sequences were generated using the Maximum-likelihood method implemented in RAxML (Stamatakis, 2014). RAxML analysis was performed using the GTRGAMMA model and 1,000 bootstrap iterations. Trees were then visualized using FigTree^1^. Trees were manually inspected and sequences forming a monophyletic group with a type strain sequence were considered to be in the same genus as the type strain.

### Evaluation of Published 16S rRNA Gene-Targeted PCR Primers for HCB Taxa

All of the sequences that were part of OTUs manually identified as belonging to HCB genera were compiled. A comprehensive list of 449 published primer sets targeting the 16S rRNA gene of *Bacteria* (Klindworth et al., 2013) was then tested against these sequences to evaluate primer coverage for HCB taxa *in silico*. Primer pairs were evaluated for perfect matches to target sequences using the R package Biostrings (Pages et al., 2014). Resulting sequences from targeted regions of the 16S rRNA gene were extracted and used for classification testing. The Ribosomal Database Project naïve Bayesian Classifier (NBC) (Wang et al., 2007), which included a 16S rRNA gene training set (v. 15), was used to taxonomically classify sequences of target taxa. Sequences were de-replicated to remove non-unique sequences and were classified using the NBC using default parameters. Primer pairs that had at least 95% coverage for targeted HCB taxa were then re-evaluated for their coverage for all *Bacteria* using TestPrime (Klindworth et al., 2013).

## Results and Discussion

### Developing a Manually Curated Database of 16S rRNA Genes Sequences of HCB Taxa

In the present study, we performed a phylogenetic analysis to validate the classification of 16S rRNA gene sequences for eight HCB taxa that are recognized as key players in the biodegradation of oil hydrocarbons in the marine environment. In order to select sequences for phylogenetic analysis, 16S rRNA gene sequences that were classified as belonging to either a family or order of the included HCB taxa (*Alteromonadaceae* [6,252 sequences], *Oceanospirillales* [23,656 sequences], *Piscirickettsiaceae* [1,571 sequences], and *Rhodospirillaceae* [5,168 sequences]) were extracted from the SILVA SSU rRNA database. These sequences were clustered into OTUs at 97% sequence identity and representative sequences were selected for each OTU (*Alteromonadaceae* [476 sequences], *Oceanospirillales* [1,577 sequences], *Piscirickettsiaceae* [228 sequences], and *Rhodospirillaceae* [1,206 sequences]) (Supplementary Data Sheet S2). In addition, 16S rRNA gene sequences from cultured representatives of these groups were extracted from the SILVA LTP database. After pre-processing of sequences, phylogenetic trees were produced and manually inspected. Generally, the taxonomic classification provided in the SILVA SSU 123 NR database was consistent with monophyletic groupings in our phylogenetic reconstruction. The HCB genus *Marinobacter* within the family *Alteromonodaceae* clustered into a well-supported monophyletic group (bootstrap support (BS) = 94) and 151 sequences were selected from this clade (Supplementary Figure S1). Within the family *Piscirickettsiaceae*, the genus *Cycloclasticus* also formed a well-supported clade (BS = 100) and 24 sequences were selected (Supplementary Figure S2). Within the family *Rhodospirillaceae*, the genus *Thalassospira* formed a well-supported clade (BS = 69) (Supplementary Figure S3). However, members of the genus *Terasakiella* formed a distinct lineage within *Thalassospira* (BS = 69, 7 sequences). These sequences were removed and the remaining 76 *Thalassospira* sequences were selected. Within the order *Oceanospirillales*, four *Oleispira* sequences were selected (BS = 92), 10 *Neptunomonas* sequences were selected (BS = 48), three *Thalassolituus* sequences were selected (BS = 60), and a single *Oleiphilus* sequence was selected (Supplementary Figure S4). *Oleiphilus* is rarely detected in sequencing surveys, and may have a restricted distribution in the environment and present only at certain sites such as the coast of Messina, Italy (Golyshin et al., 2002). The genus *Alcanivorax* had low bootstrap support (BS = 8). We therefore opted for a conservative approach and only lineages with cultured representatives were selected, resulting in 135 selected sequences for this genus. All selected sequences are available in the Supplementary Information.

### Selecting Candidate 16S rRNA Gene-Targeted PCR Primer Pairs to Detect HCB

**FIGURE 2:**
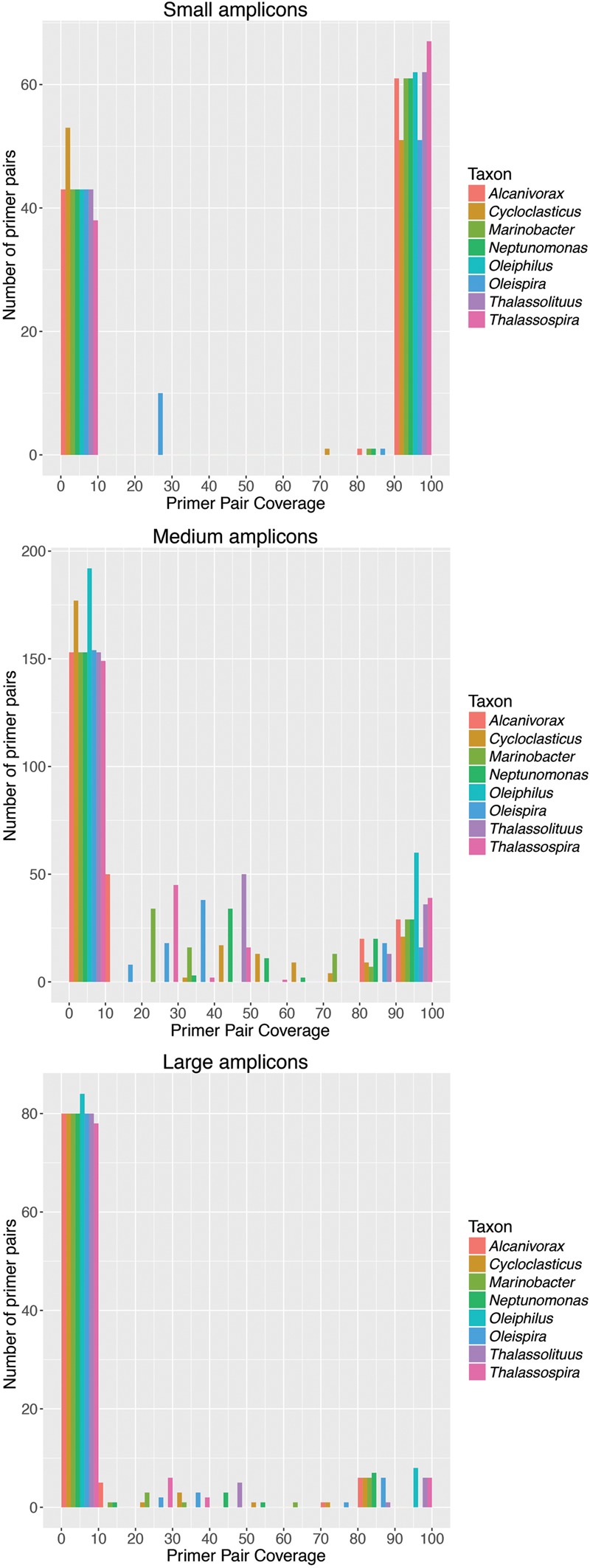
**Large variability in coverage of universal bacterial 16S rRNA gene primer pairs for HCB taxa.** Histograms are shown of coverage for primer pairs designed to produce amplicons of either small (100-400 bp; 105 primer pairs), medium (>400–<1000 bp; 252 primer pairs), or large (>1000 bp; 92 primer pairs) size. The y-axis of the histograms shows the number of primer pairs identified for each interval of coverage, which has been divided into intervals of 10%. Coverage is shown for each target taxon separately to illustrate the variability between coverage across target taxa. Many primer pairs in each of the amplicon size ranges have <10% coverage for at least one target taxa. Primer pairs were identified with >90% coverage for at least one target taxon for all amplicon size ranges, though no large amplicon primer pairs captured the taxa *Alcanivorax, Cycloclasticus*, or *Oleispira* with >90% coverage. Details for each primer pair are given in **Supplementary Table S1**.

Using the validated set of HCB taxa sequences, including all sequences within each OTU, we evaluated a comprehensive list of published universal *Bacteria* 16S rRNA gene-targeted primer pairs for their coverage for these taxa (**Supplementary Table S1**). We used the grouping of primer pairs into small (100–400 bp), medium (>400–<1000 bp), and large (>1000 bp) amplicons according to Klindworth et al. (2013) and evaluated perfect-match coverage for target taxa *in silico*. For all three amplicon size classes, there was a large distribution of coverage of primer pairs, with many primer pairs having less than <10% coverage for some or all of the target taxa (**Figure 2**). Generally, more primer pairs producing short amplicons had high coverage (>90%) than primer pairs producing medium or long amplicons. An optimal primer pair would have high coverage for each of the eight HCB target taxa, but we observed large variability in the number of primer pairs with high coverage across all of the target taxa. For each amplicon size category, primer pairs were identified having at least 83–95% coverage (**Figure 2** and **Supplementary Table S1**; Minimum coverage for each target taxon: short amplicon = 93.8%, medium amplicon = 94.7%, long amplicon = 83.4%). Multiple short and medium amplicon primer pairs were identified that had >90% coverage for each of the target taxa, though no long amplicon primer pairs were identified with >90% coverage for each of the target taxa (**Figure 2** and **Supplementary Table S1**; short amplicon = 51 out of 105 pairs; medium amplicon = 16 out of 252 pairs; long amplicon = 0 out of 92 pairs). The reason for this is that no large amplicon primer pairs had >90% coverage for the groups *Alcanivorax, Cycloclasticus*, and *Oleispira* (**Figure 2**). These results highlight that if long-read sequencing technologies become practical for amplicon sequencing new primers should be designed to better target these taxa. A list of primer pairs with >95% coverage for all target taxa can be found in **Supplementary Table S1**. We re-evaluated the coverage of these primer pairs for all *Bacteria* and found that almost all had >80% coverage, indicating that they are all useful for capturing general bacterial diversity as well as obligate HCB taxa. We note that for this comparison we made a conservative assumption by considering only perfect matches to target sequences. Allowing for mismatches would be expected to generally increase coverage values.

Currently, most amplicon sequencing is performed using the Illumina MiSeq platform, which is amenable to medium-length amplicons. We therefore selected the primer pair with the highest coverage in this group for further analysis. The primer pair, S-D-Bact-0343-a-S-15 (forward) and S-D-Bact-0908-a-A-18 (reverse) forms an amplicon of 583 bp and covers the hyper-variable regions 3–5. In order to determine if amplicons from target taxa produced from this primer pair would be correctly classified using a standard classification analysis, we extracted *in silico*-derived amplicons from sequences of target taxa to be classified with the RDP näive Bayesian classifier. This analysis revealed that almost all (≥97% for all groups) target amplicons were correctly classified to the genus level, and correctly classified sequences were almost all classified with ≥80% confidence (**Supplementary Table S2**).

## Conclusion

We found that universal bacterial 16S rRNA gene primer sets varied widely in their coverage for the eight HCB taxa evaluated here. This suggests that differences in HCB prevalence across different studies must be interpreted with caution and highlights the need for careful primer selection. There was no consistent trend for any single genus to be missed by all primers, but few primer pairs covered the genera *Oleiphilus* and *Thalassolituus*. Poor primer coverage may also help to explain the rare detection of *Oleiphilus.* Generally, many primer pairs are available that have high overall coverage of HCB taxa for small and medium sized amplicons, but coverage is more limited for primers designed to produce large amplicons. For medium-sized amplicons amenable to the currently widely used Illumina MiSeq platform, we identified the primer pair S-D-Bact-0343-a-S-15 (forward) and S-D-Bact-0908-a-A-18 (reverse) with the highest overall coverage, and which targeted all groups with at least 95% coverage. We therefore recommend the use of this primer pair for studies focused on detection of these obligate HCB in the environment. Though *in silico* analysis suggests that all primer pairs are compatible for PCR, experimental testing would be necessary before employing any new primer pair. In future work, we aim to explore this and other primer pairs to identify also ‘generalist’ hydrocarbon degraders.

## Author Contributions

DB and TG designed the study, analyzed the results, and wrote the manuscript. DB performed computational experiments.

## Conflict of Interest Statement

The authors declare that the research was conducted in the absence of any commercial or financial relationships that could be construed as a potential conflict of interest.

## Footnotes

1 http://tree.bio.ed.ac.uk/software/figtree

## Supplementary Material

TABLE S1 **Details and coverages of 16S rRNA gene primer sets**.

TABLE S2 **Classification results for validated sequences using the region amplified by primers S-D-Bact-0343-a-S-15 and S-D-Bact-0908-a-A-18 (HV regions 3-5).** Amplicons were classified with RDP Classifier 16S rRNA database version 15. When stringent confidence thresholds are used, the number of “true positive” classifications decreases (rightmost column).

## References

[B1] Edgar R. C. (2010). Search and clustering orders of magnitude faster than BLAST. *Bioinformatics* 26 2460–2461. 10.1093/bioinformatics/btq461 20709691

[B2] Golyshin P. N. Chernikova T. N. Abraham W.-R. Lünsdorf H. Timmis K. N. Yakimov M. M. (2002). *Oleiphilaceae* fam. nov., to include *Oleiphilus messinensis* gen. nov., sp. nov., a novel marine bacterium that obligately utilizes hydrocarbons. *Int. J. Syst. Evol. Microbiol.* 52 901–911. 12054256 10.1099/00207713-52-3-901

[B3] Gutierrez T. Green D. H. Nichols P. D. Whitman W. B. Semple K. T. Aitken M. D. (2012a). *Algiphilus aromaticivorans* gen. nov., sp. nov., an aromatic hydrocarbon-degrading bacterium isolated from a culture of the marine dinoflagellate *Lingulodinium polyedrum*, and proposal of *Algiphilaceae* fam. nov. *Int. J. Syst. Evol. Microbiol.* 62 2743–2749. 10.1099/ijs.0.033324-0 22228670

[B4] Gutierrez T. Green D. H. Whitman W. B. Nichols P. D. Semple K. T. Aitken M. D. (2013a). *Polycyclovorans algicola* gen. nov., sp. nov., an aromatic hydrocarbon-degrading marine bacterium found associated with laboratory cultures of marine phytoplankton. *Appl. Environ. Microbiol.* 79 205–214. 10.1128/AEM.02833-12 23087039 PMC3536080

[B5] Gutierrez T. Nichols P. D. Whitman W. B. Aitken M. D. (2012b). *Porticoccus hydrocarbonoclasticus* sp. nov., an aromatic hydrocarbon-degrading bacterium identified in laboratory cultures of marine phytoplankton. *Appl. Environ. Microbiol.* 78 628–637. 10.1128/AEM.06398-11 22139001 PMC3264135

[B6] Gutierrez T. Singleton D. R. Berry D. Yang T. Aitken M. D. Teske A. (2013b). Hydrocarbon-degrading bacteria enriched by the Deepwater Horizon oil spill identified by cultivation and DNA-SIP. *ISME J.* 7 2091–2104. 10.1038/ismej.2013.98 23788333 PMC3806270

[B7] Hazen T. C. Dubinsky E. A. DeSantis T. Z. Andersen G. L. Piceno Y. M. Singh N. (2010). Deep-sea oil plume enriches indigenous oil-degrading bacteria. *Science* 330 204–208. 10.1126/science.1195979 20736401

[B8] Head I. M. Jones D. M. Röling W. F. M. (2006). Marine microorganisms make a meal of oil. *Nat. Rev. Microbiol.* 4 173–182. 10.1038/nrmicro1348 16489346

[B9] Joye S. B. Teske A. P. Kostka J. E. (2014). Microbial dynamics following the Macondo oil well blowout across Gulf of Mexico environments. *BioScience* 64 766–777.

[B10] Kimes N. E. Callaghan A. V. Aktas D. F. Smith W. L. Sunner J. Golding B. (2013). Metagenomic analysis and metabolite profiling of deep-sea sediments from the Gulf of Mexico following the Deepwater Horizon oil spill. *Front. Microbiol.* 4:50 10.3389/fmicb.2013.00050 PMC3598227 23508965

[B11] Klindworth A. Pruesse E. Schweer T. Peplies J. Quast C. Horn M. (2013). Evaluation of general 16S ribosomal RNA gene PCR primers for classical and next-generation sequencing-based diversity studies. *Nucleic Acids Res.* 41 e1 10.1093/nar/gks808 PMC3592464 22933715

[B12] Pages H. Aboyoun P. Gentleman R. DebRoy S. (2014). *Biostrings: String Objects Representing Biological Sequences, and Matching Algorithms*. R package version at least 2.32.0.

[B13] Prince R. C. Gramain A. McGenity T. J. (2010). “Prokaryotic hydrocarbon degraders,” in *Handbook of Hydrocarbon and Lipid Microbiology* eds Timmis K. N. McGenity T. J. van der Meer J. R. de Lorenzo V. (Berlin: Springer) 1671–1692.

[B14] Quast C. Pruesse E. Yilmaz P. Gerken J. Schweer T. Yarza P. (2013). The SILVA ribosomal RNA gene database project: improved data processing and web-based tools. *Nucleic Acids Res.* 41 D590–D596. 10.1093/nar/gks1219 23193283 PMC3531112

[B15] Schloss P. D. Westcott S. L. Ryabin T. Hall J. R. Hartmann M. Hollister E. B. (2009). Introducing mothur: open-source, platform-independent, community-supported software for describing and comparing microbial communities. *Appl. Environ. Microbiol.* 75 7537–7541. 10.1128/AEM.01541-09 19801464 PMC2786419

[B16] Stamatakis A. (2014). RAxML version 8: a tool for phylogenetic analysis and post-analysis of large phylogenies. *Bioinformatics* 30 1312–1313. 10.1093/bioinformatics/btu033 24451623 PMC3998144

[B17] Wang Q. Garrity G. M. Tiedje J. M. Cole J. R. (2007). Naive Bayesian classifier for rapid assignment of rRNA sequences into the new bacterial taxonomy. *Appl. Environ. Microbiol.* 73 5261–5267. 10.1128/AEM.00062-07 17586664 PMC1950982

[B18] Yakimov M. M. Timmis K. N. Golyshin P. N. (2007). Obligate oil-degrading marine bacteria. *Curr. Opin. Biotechnol.* 18 257–266. 10.1016/j.copbio.2007.04.006 17493798

[B19] Yang T. Nigro L. M. Gutierrez T. D’Ambrosio L. Joye S. B. Highsmith R. (2016). Pulsed blooms and persistent oil-degrading bacterial populations in the water column during and after the Deepwater Horizon blowout. *Deep Sea Res. Part II Top. Stud. Oceanogr.* 129 282–291. 10.1016/j.dsr2.2014.01.014

[B20] Yarza P. Richter M. Peplies J. Euzeby J. Amann R. Schleifer K. H. (2008). The All-Species Living Tree project: a 16S rRNA-based phylogenetic tree of all sequenced type strains. *Syst. Appl. Microbiol.* 31 241–250. 10.1016/j.syapm.2008.07.001 18692976

