# Supplementary material for: Evaluating the Detection of Hydrocarbon-Degrading Bacteria in 16S rRNA Gene Sequencing Surveys
Source: Front Microbiol. 2017 May 17;8:896. doi: 10.3389/fmicb.2017.00896 (PMC5434106; doi:10.3389/fmicb.2017.00896)
Supplement: TABLE S2 — Classification results for validated sequences using the region amplified by primers S-D-Bact-0343-a-S-15 and S-D-Bact-0908-a-A-18 (HV regions 3-5). Amplicons were classified with RDP Classifier 16S rRNA database version 15. When stringent confidence thresholds are used, the number of “true positive” classifications decreases (rightmost column). [file Data_Sheet_1.DOCX]

**Supplemental Figures and Tables**


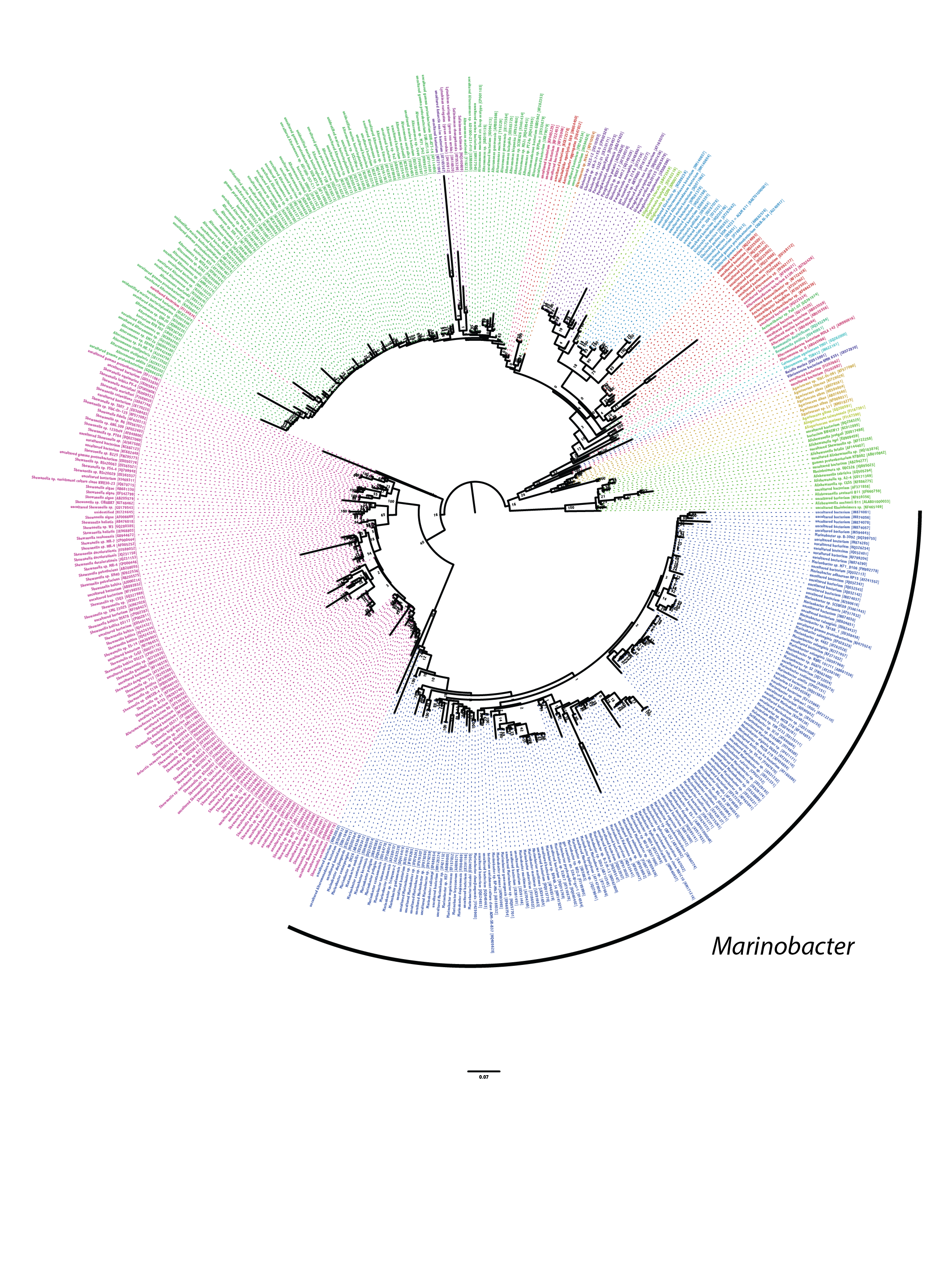


**Figure S1.** Maximum-likelihood tree of 16S rRNA gene sequences of the family *Alteromonadaceae*. The tree is colored by the taxonomic classification in the SILVA SSU Ref 123 database. Target genera are labeled. Bar indicates 7 substitutions per 100 nucleotide positions.


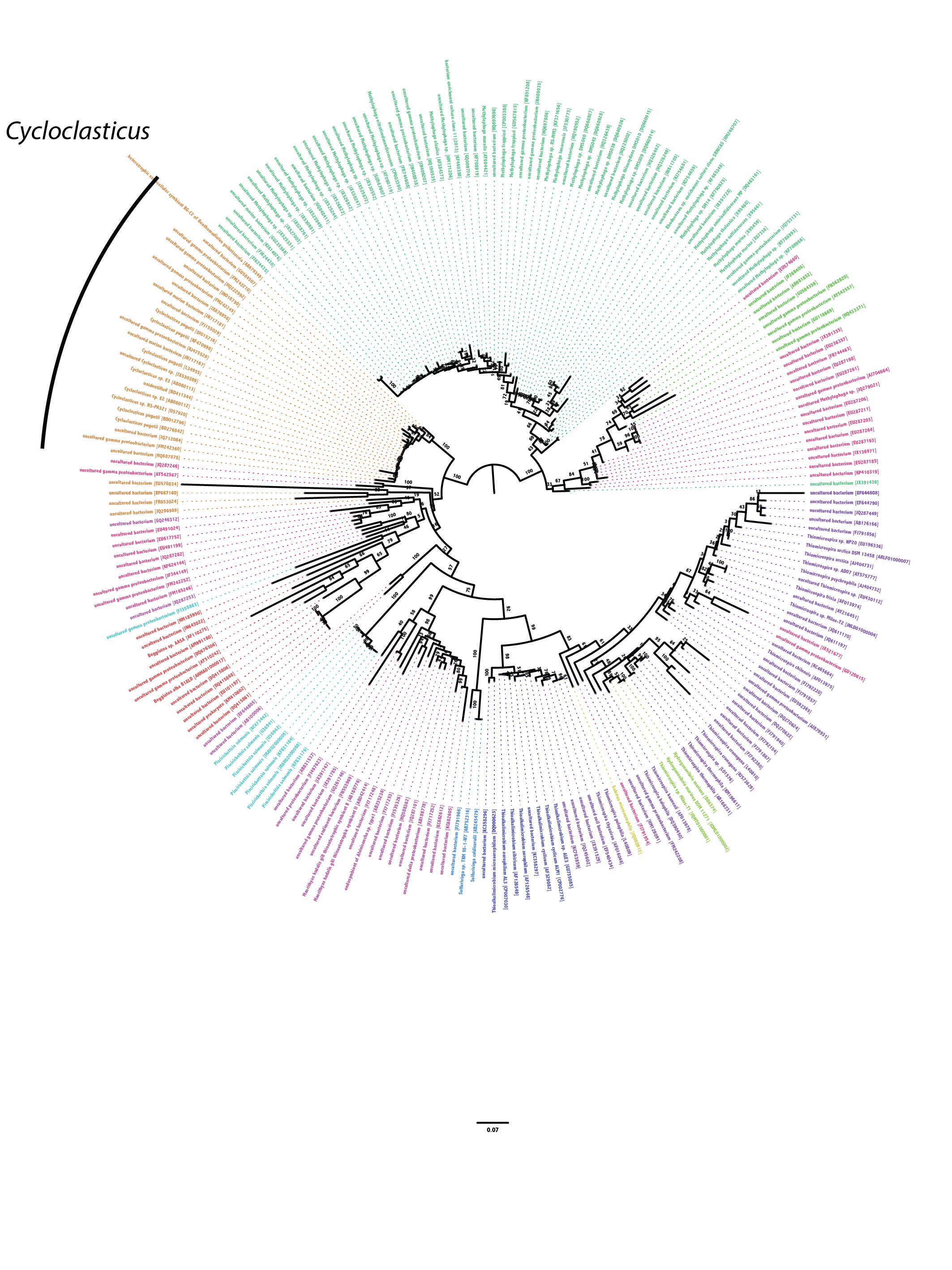


**Figure S2.** Maximum-likelihood tree of 16S rRNA gene sequences of the family *Piscirickettsiaceae*. The tree is colored by the taxonomic classification in the SILVA SSU Ref 123 database. Target genera are labeled. Bar indicates 7 substitutions per 100 nucleotide positions.


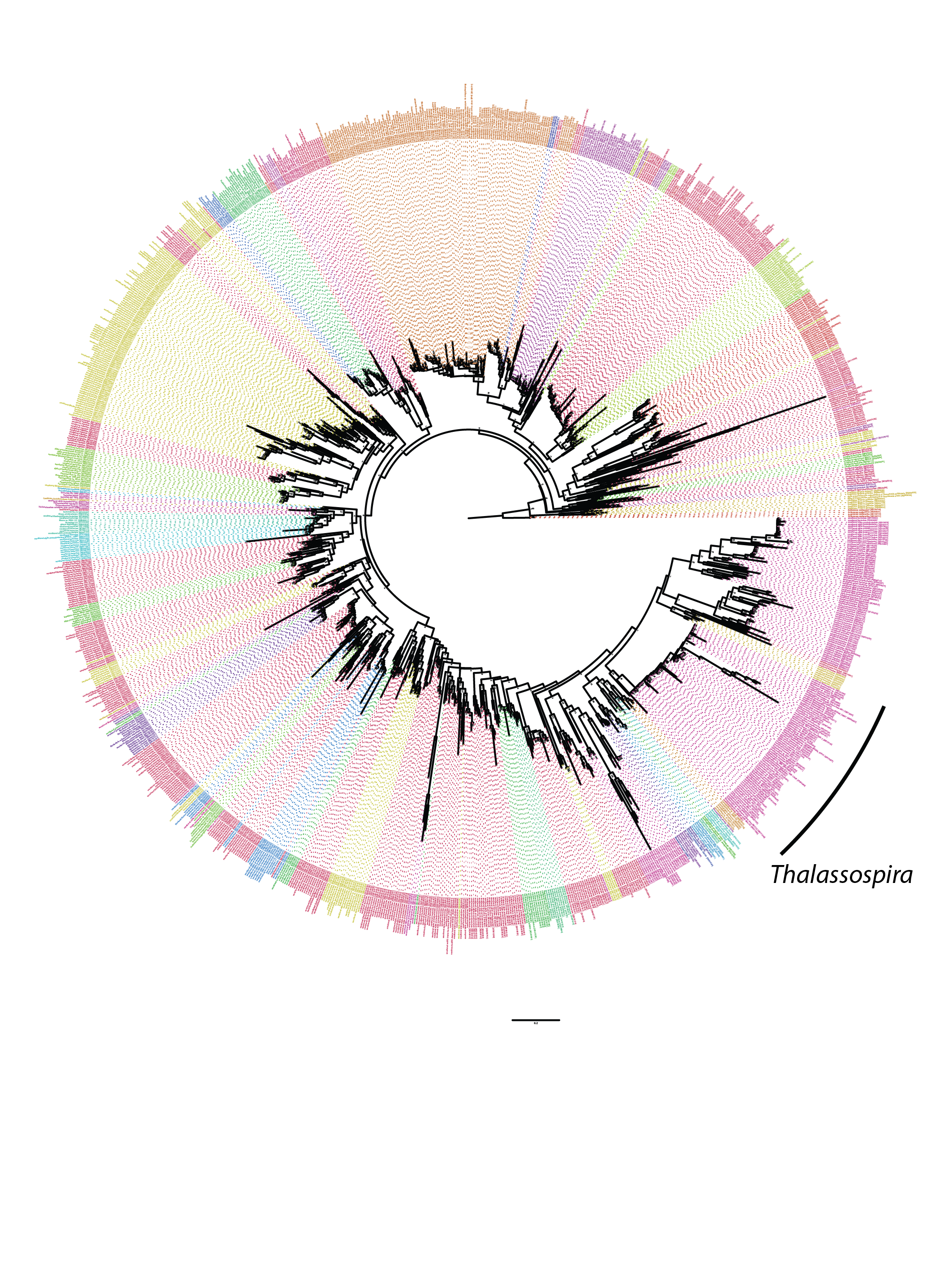


**Figure S3.** Maximum-likelihood tree of 16S rRNA gene sequences of the family *Rhodospirillaceae*. The tree is colored by the taxonomic classification in the SILVA SSU Ref 123 database. Target genera are labeled. Bar indicates 7 substitutions per 100 nucleotide positions.


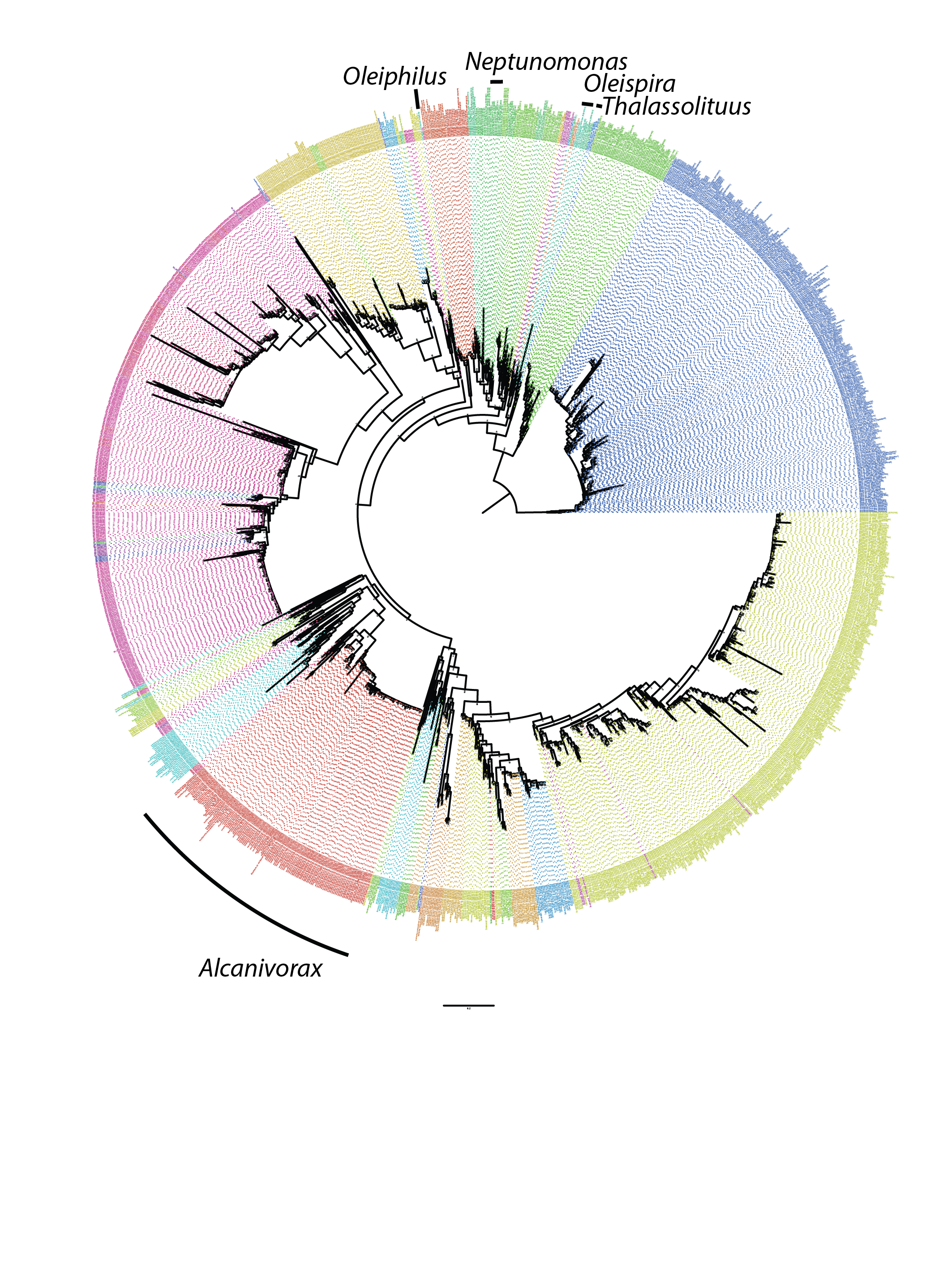


**Figure S4.** Maximum-likelihood tree of 16S rRNA gene sequences of the order *Oceanospirillales*. The tree is colored by the taxonomic classification in the SILVA database. Target genera are labeled. Bar indicates 20 substitutions per 100 nucleotide positions.

**Table S2**. Classification results for validated sequences using the region amplified by primers S-D-Bact-0343-a-S-15 and S-D-Bact-0908-a-A-18 (HV regions 3-5). Amplicons were classified with RDP Classifier 16S rRNA database version 15. When stringent confidence thresholds are used, the number of “true positive” classifications decreases (rightmost column).

| Genus | No. Tested Seqs. | % Correctly classified at any confidence level | % Correctly classified and with classification >=80% confidence |
| --- | --- | --- | --- |
| *Alcanivorax* | 732 | 100 | 98 |
| *Cycloclasticus* | 61 | 100 | 100 |
| *Marinobacter* | 749 | 99.6 | 99.8 |
| *Neptunomonas* | 38 | 100 | 100 |
| *Oleiphilus* | 1 | 100 | 100 |
| *Oleispira* | 35 | 100 | 100 |
| *Thalassolituus* | 19 | 100 | 100 |
| *Thalassospira* | 116 | 97 | 100 |
